# Patient-reported negative experiences related to caries and its treatment among Swedish adult patients

**DOI:** 10.1186/s12903-017-0384-3

**Published:** 2017-06-05

**Authors:** Håkan Flink, Åke Tegelberg, Judith E. Arnetz, Dowen Birkhed

**Affiliations:** 1 Centre for Clinical Research, Uppsala University, Västmanland County, Västerås, Sweden; 2 Public Dental Clinic, Public Dental Health Västmanland, Sala, Sweden; 3 Faculty of Odontology, Malmö University, Malmö, Sweden; 4 Postgraduate Dental Education Center, Public Dental Service, Örebro, Sweden; 5 Department of Public Health and Caring Sciences, Uppsala University, Uppsala, Sweden; 6 Department of Family Medicine, College of Human Medicine, Michigan State University, East Lansing, MI USA; 7 Fersens väg, Malmö, Sweden

**Keywords:** Attitudes, Dental caries, Dental records, Long-term evaluation, Negative emotions, Negative experiences, Questionnaire

## Abstract

**Background:**

It has been suggested that dental caries should be regarded as a chronic disease as many individuals repeatedly develop new caries lesions. How this is perceived by caries active patients is unclear. The aim of this study was to measure patient-reported attitudes and negative experiences related to caries and dental treatment.

**Methods:**

A questionnaire was mailed to 134 caries active (CA) and 40 caries inactive (CI) adult patients treated at a Swedish public dental service clinic. The questionnaire included items regarding patient-reported oral health; attitudes towards caries and efforts to prevent them; and negative experiences related to caries and dental treatment. Questionnaire data were supplemented with data on caries and caries prophylaxis from patients’ dental records. Exploratory factor analysis was conducted on items related to patients’ perceptions of problems to see whether scales could be created. Experiences, perceptions and dental records of CA and CI patients were compared.

**Results:**

The overall response rate was 69%. Dental records confirmed that CA patients had significantly more decayed teeth per year and a longer period of caries-active time than CI patients. Factor analysis resulted in 3 distinct scales measuring problems related to caries; 1) caries-related information; 2) negative experiences; and 3) negative treatment/staff attitudes. A fourth scale measuring perceived problems related to caries was also created. The CA group reported significantly more problems related to caries and dental treatment, received significantly more caries-related information, and reported significantly more negative treatment experiences compared to CI patients.

**Conclusions:**

Caries prophylaxis methods need to be improved in order to better meet the needs of caries active patients and to create a more positive experience with dental care.

**Electronic supplementary material:**

The online version of this article (doi:10.1186/s12903-017-0384-3) contains supplementary material, which is available to authorized users.

## Background

It has been proposed that dental caries in most patients should be regarded as a more or less “chronic disease” [1, 2]. Yet, several of the caries preventive regimens that are used today are based on limited scientific evidence [3, 4]. Most studies regarding caries prophylaxis have been performed on children and teenagers [3, 4], and a majority of them have been conducted without accounting for the individual’s caries risk [3, 4]. When a high risk strategy has been employed among children, the results indicate that many of the methods used today do not decrease caries progression [5, 6]. Thus, there is a lack of evidence for caries preventive methods in adults with increased caries risk. In general practise, the caries prophylaxis actions also tend to be occasional and more random rather than following structured programs or strategies [7, 8].

Knowledge regarding how development of new caries lesions is perceived by caries active individuals in relation to dental treatment is incomplete, even if some problems have been described [9, 10]. Among adolescents and teenagers, it is important to give adequate information on caries risk since this patient group may display a rather passive attitude, i.e. “everything will be all right and fixed by the dentist”, or even a negative attitude, characterized by frustration and a tendency to give up [9].

Among adults, toothache and decayed and filled teeth are correlated with fear of dental treatment and inversely related to satisfaction with dental care [10]. Longitudinal caries studies among adults are rare, but available data indicate that caries active individuals continue to be caries active for many years [11, 12]. Therefore, it could be presumed that continuing caries activity for many years may perpetuate and even increase the negative experiences of dental care.

The aim of the present investigation was to measure patient-reported problems and negative experiences related both to caries and to dental treatment and to compare caries active and caries inactive individuals in these respects in one and the same clinic.

## Methods

### Setting and participants

The study was performed at the public dental service clinic in the municipality of Sala, Sweden. A total of 134 caries active (CA) and 40 caries inactive (CI) individuals were recruited during 2007 and have been presented in an earlier study [13]. Among these, 174 participants, 40 CA and 40 CI patients have previously been identified [14], and an additional 94 CA patients 25–50 years of age were consecutively recruited.

The following definitions of the two groups were used: “The CA group” included individuals who had developed manifest primary or secondary caries lesions in 2 or more teeth in the last 3 years. “The CI group” was individuals who had been free from manifest caries for 3 years or more. Caries prevalence among the two groups has previously been described in detail [13]. There were statistically significant differences between the CA and CI groups for all caries-related variables, such as number of decayed teeth (DT), root filled teeth, extracted teeth, decayed, missing and filled tooth surfaces (DMFS) and caries active time during the follow-up period. *“*Caries active time” was defined as the time between two examinations where the patients showed development of manifest caries. Consequently, *“*caries inactive time” was defined as the time between two examinations where no manifest caries lesions were recorded.

### Questionnaire

A questionnaire, along with detailed written information about the study, was mailed to all eligible individuals (*n* = 174) in 2007. Two reminders were sent 3 and 6 weeks later to those who did not respond to the first invitation. All participants answering the questionnaire also returned a signed consent form. The questionnaire was developed for this study and was piloted on several occasions among test groups of both caries active and inactive patients.

The questions were focused on general health, diet, oral hygiene habits, sociodemographic variables and perception of caries active time as previously described [13]. Furthermore, questions regarding caries prophylaxis [15], carried out both at the clinic and at home, were also included. The complete questionnaire used “Oral health and caries.pdf” is uploaded as “Additional file 1”.

The questions of interest in the current study concerned two parts (here called A and B): A = “patient- reported problems” and B = “patient negative experiences” related to caries and dental treatment.

(A) * ”Patient-reported problems”:* Five questions (No 1–5) measured current perceptions of situations related to caries. The question No 1 (“caries is a problem for me today”), was rated on a visual analog scale (VAS) with anchor values from 0 = no problem to 100 = very much a problem. The four additional questions (No 2–5) concerned: 2) the effect on the patient’s personal economy; 3) the time needed for visits to the dentist; 4) discomfort during the dental visits for caries treatment; and 5) trouble/pain with one’s teeth because of caries. Patients were asked to rate the degree to which they perceived each of the four questions as problematic on a four-point scale where 1 = disagree completely and 4 = agree completely.
(B) * “Patient negative experiences”:* These questions were related to dental treatment and included statements about negative emotions, treatment and attitudes of dental staff. The questions were constructed as Likert-type scales with 5 response alternatives, ranging from “never” to “very often.” Twenty questions concerned information and recommendations regarding caries that the patient had received, and negative emotional responses that a patient may have had during dental treatment, including whether the patient had experienced pain or felt afraid, stressed, or anxious. Negative treatment and attitudes were measured by four questions, one example was: “How often, as an adult, have you been treated in a condescending manner?” An example of a question related to information was: “How often have you, as an adult, been informed that you have caries?” An example related to negative experiences was: “How often have you, as an adult, experienced that your dental treatment was painful?”

### Dental records

Dental records were reviewed retrospectively to the patient age of 20 years or as far back as possible. Theoretically, this would provide a minimum follow up period of 5 years among the youngest participants. Information regarding caries prevalence and caries prophylaxis measures was registered. There were statistically significant differences between the two groups for all caries-related variables, such as number of decayed teeth (DT), root filled teeth, extracted teeth, decayed, missing and filled tooth surfaces (DMFS) and “caries active time” during the follow-up period [13]. The CA individuals had received more information and recommendations about caries and caries prophylaxis than the CI individuals, and had also made more extra caries prophylaxis efforts at home. However, 60% of the CA individuals had not experienced that they had become free from caries (i.e. not needing fillings) when evaluating the effect of the extra caries prophylaxis efforts that they had performed. This was confirmed by data from the dental records [15].

### Statistical methods

Differences between the two groups were tested by t-tests for continuous variables and by chi-square test for categorical variables. All tests were two-sided and *p*-values less than 0.05 were considered significant. . Based on previous research with similar populations we expected approximately 33% of the patient population to be caries active [16]. We intended to test the hypothesis that the caries active and inactive groups varied in dental treatment and patient-reported attitudes and negative experiences via a series of two-tailed t-tests. In order to detect a medium effect size (Cohen’s d = .65), expected in patient-reported outcomes research of this type [17], with a power of .80 and a p-value of .05, a sample size of 77 for the caries inactive group and 25 for the caries active group was required (total *n* = 102). Regarding patient dental records, calculation of sample size was based on data from a pilot sample, where the caries active group had received a mean number of 1.5 basic prophylaxis activities per year while the caries inactive group received 0.4 activities.

To detect an expected difference of 1.1 mean number of basic prophylaxis activities per year with a power of 80% and a significance level of 5%, assuming a standard deviation of 1.5, it would require a sample size of 60 persons plus 30% drop outs.

### Scale construction and factor analysis

The questions related to the patient’s perceptions of problems (*n* = 5) and negative experiences and emotions (*n* = 20) had not been previously validated. Therefore, for each group of questions a correlation matrix was created to study the inter-item correlations and to see whether any scales could be created. Bartlett’s test of sphericity and the Kaiser-Meyer-Olkin measure of sampling adequacy (KMO) were used to assess the factorability of the correlation matrix. KMO values should be close to 1, with a suggested minimum value of 0.6 [18]. Exploratory factor analysis was used to examine the structure of relationships between the items and to examine whether scales could be created. Principal components analysis using varimax rotation and scree plots was used to extract the factors. Internal reliability of the scales was measured using Cronbach’s alpha. Criteria for scale creation were that each scale should have (1) clinical relevance based on face validity; (2) item loadings of 0.30 or higher [19]; (3) a minimum of 3 items; and (4) a reliability coefficient of 0.70 or greater. Although there is disagreement as to sample size requirements for factor analysis [20, 21], a minimum sample size of 100 is recommended and/or a subject-to-variables (STV) ratio of approximately 5 [22]. Our sample size of 120 fulfilled both of these criteria.

All analyses were conducted using SPSS version 20.0, Chicago, IL, USA.

## Results

**Fig. 1:**
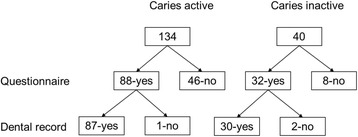
Flow chart showing eligible individuals. Number receiving questionnaire, number of returned questionnaires and number of retrieved dental records

The overall response rate to the postal questionnaire was 69% (120/174). Of these 120 patients, complete dental records could be obtained for 87 out of the 88 in the CA and 30 out of the 32 in the CI group (Fig. 1). A comparison of background characteristics between the two groups is summarized in Table 1; they did not differ significantly by gender, age, follow-up time, number of examinations or the number of dentists treating them. Most of the dentists treated both the CA and CI patients. The CA patients had significantly more decayed teeth (DT) per year and longer period of caries active time than the CI patients (*p* < 0.001*).*

**Table 1:** Background characteristics of the caries active (CA) and caries inactive (CI) of participating patients (for details, see references [13, 15])

	CA		CI		
	*n*	%	*n*	%	*p*
Gender					
Men	24	27	9	28	0.93
Women	64	73	23	72	
		Mean ± SD		Mean ± SD	
Age	88	39.5 ± 6.2	32	41.0 ± 6.3	0.27
Follow up time of dental records	87	16.5 ± 6.8	30	18.3 ± 6.4	0.22
Caries active time	87	9.9 ± 4.7	30	1.7 ± 2.1	<0.001
Caries active time/ Follow up time	87	0.6 ± 0.2	30	0.1 ± 0.1	< 0.001
DT per year	87	1.0 ± 0.6	30	0.1 ± 0.1	< 0.001
Number of examinations	87	12.3 ± 5.7	30	10.8 ± 4.2	0.18
Number of dentist performing the examinations	87	5.3 ± 2.5	30	5.1 ± 2.4	0.68

*DT* number of decayed teeth

### Factor analysis

#### Perceived problems related to caries

In factor analysis of the 5 items related to caries being perceived as a problem, the Kaiser-Meyer-Olkin (KMO) measure of sampling adequacy was 0.875 and Bartlett’s test of sphericity was significant (Chi-square = 310, *p* = .000), justifying proceeding with the analysis. The five items did load on a single factor, explaining 70% of the variance; however, Cronbach’s alpha was only 0.18. We therefore excluded the item measuring caries as a problem on the visual analogue scale, as it utilized a different response scale compared to the other 4 items. When excluding the VAS item, the KMO was 0.827, Bartlett’s test remained significant (Chi-square 243, *p* = .000), and the 4 items formed a single-factor scale with a Cronbach’s alpha of 0.87. These 4 items (Table 2) explained 73% of the variance in perceptions of caries as a problem.

**Table 2:** Question: *Have caries been a problem for you….?*

	CA		CI		
	*n*	Mean ± SD	*n*	Mean ± SD	*p*
a/…for your economic situation?	85	2.33 ± 1.07	32	1.44 ± 0.80	< 0.001
b/…causing trouble/pain from your teeth?	84	2.24 ± 0.99	32	1.22 ± 0.61	< 0.001
c/…causing inconvenience during treatment at the dentist?	84	2.00 ± 0.89	32	1.19 ± 0.59	< 0.001
d/…due to the time you spent at the dentist for caries treatment?	83	1.88 ± 0.76	32	1.09 ± 0.30	< 0.001

When evaluating problems related to caries on a VAS scale, significantly higher values were found in the CA than in the CI group, 62 ± 24 compared to 12 ± 15 (*p* < 0.001*).* When examining more specific questions, each problem was experienced significantly more often among CA patients, compared to the CI group (Table 2).

#### Perceived negative experiences

Exploratory factor analysis of the 20 experience items resulted in the extraction of 4 factors with eigenvalues >1, explaining 66% of the total variance. Bartlett's test of sphericity was significant (Chi-square = 1100, df = 153, *p* < 0.001) and KMO was 0.86, which justified proceeding with the factor analysis. Four items concerning specific treatments or care instructions that patients may have received did not load on any of the factors and were therefore excluded from subsequent analyses. Factor analysis of the remaining 16 items resulted in the extraction of 3 factors with eigenvalues >1, explaining 66% of the total variance. One item concerning whether the patient had been recommended to reduce the number of snacks that they eat showed shared variance between two of the 3 factors and was therefore excluded. Table 3 summarizes the results of the exploratory factor analysis of the remaining 15 items related to patient experiences in conjunction with dental visits. Based on the item structure suggested by the exploratory factor analysis and item content, the 3 factors were named Caries-related information (6 items), Negative experiences/emotions (6 items), and Negative treatment/attitudes (3 items). Cronbach’s alphas for each of the 3 scales were acceptable (Table 3).

**Table 3:** Loadings of variables on factors (bold print) emerging from rotated component matrix with reliability estimates (Cronbach’s alpha)

Item nr.	Abbreviated item label	Factor 1 Caries-related information	Factor 2 Negative experience/emotions	Factor 3 Negative treatment/attitudes
1	Told you had caries	**0.78**	0.28	0.09
2	Told you needed extra caries-preventive treatment	**0.86**	0.27	0.09
3	Informed about causes of caries	**0.80**	0.20	0.03
5	Recommended limiting your intake of sugar	**0.69**	0.13	0.24
6	Recommended using some form of fluoride	**0.84**	0.15	0.14
11	Told your immunity to caries was impaired	**0.79**	0.00	0.26
12	Treatment was painful	0.31	**0.70**	0.02
13	You felt calm^a^	0.09	**0.81**	0.16
14	You felt frightened	0.23	**0.84**	0.11
15	You felt stressed	0.23	**0.72**	0.32
16	You felt anxious	0.11	**0.86**	0.16
17	You felt you were in control^a^	0.07	**0.48**	0.16
18	You were treated in a condescending manner	0.16	0.11	**0.69**
19	You were disappointed	0.10	0.25	**0.81**
20	You felt powerless	0.29	0.29	**0.78**
Cronbach’s alpha		0.91	0.87	0.75

^a^These items were reverse-scored

**Item excluded from analyses because shared variance between Factor 1 and 2**

4 Recommended to reduce the number of snacks between meals

**Item excluded from analyses as they did not load on any of the factors**

7 Had your teeth polished

8 Had fluoride varnish applied on your teeth

9 Instructed how to brush your teeth

10 Instructed how to use dental floss or other devices to clean between your teeth

#### Scale scores

Mean values were calculated for each of the four scales, with higher scores indicating more problems, receipt of more caries-related information, and more negative experiences and treatment/attitudes. A comparison of mean scores for the CA and CI groups using independent t-tests revealed significantly higher problem, caries-related information, and negative experience scores for the CA group (Table 4).

**Table 4:** Comparison of scale scores, caries active (CA, *n* = 88) and caries inactive (CI, *n* = 32)

Scale	Group	*n*	Mean	t	*p*-value
Problems related to caries^a^	CA	85	2.12	7.73	<.001
	CI	32	1.23		
Caries-related information	CA	87	2.86	12.49	<.001
	CI	32	1.51		
Negative experience/emotions	CA	87	2.51	3.16	<.01
	CI	32	1.97		
Negative treatment/attitudes	CA	87	1.70	5.33	<.001
	CI	31	1.20		

^a^Score range 1–4. For all other scales, score range is 1–5

## Discussion

This study examined patient-reported problems and negative experiences related to caries and dental treatment and compared responses between caries active and caries inactive individuals. The CA group reported significantly more problems related to caries and dental treatment and had received more caries-related information. They also reported more negative emotions related to caries and dental treatment as well as having been met by negative attitudes from dental staff.

The CA group reported significantly more economic problems and discomfort from their teeth, even if the question used “trouble/pain with one’s teeth because of caries” has limitations regarding the patient’s ability to describe the amount and severity of the trouble/pain and if it is really caused by caries. Nevertheless, these perceptions might correspond with the observation that individuals having refrained from dental treatment for financial reasons also reported poorer self-rated oral health [23]. However, in this sample the CA group had a shorter mean recall interval than the CI group which can be interpreted as no or very few of the participants had refrained from dental treatment for financial reasons [13], in spite the fact that they have answered that caries have been a problem for their economy. Toothache and decayed and filled teeth have also been correlated with utilization of dental care, and with a negative effect on satisfaction with dental care [10]. A higher restorative rate has also been reported to have an inverse association to satisfaction with oral health [24].

In the present study, we did not ask specifically about satisfaction with dental care or treatment, but we theorize that patients reporting significantly more negative emotions related to caries and dental treatment among the CA group would be less satisfied compared with the CI group. The difference in negative emotions likely corresponds to the differences in need of dental treatment. Even if treatments are carried out very carefully with extensive information and generous amounts of anaesthetic, they can be painful, causing stress and anxiety and making patients fearful. Negative emotions related to dental treatment have been investigated among patients with dental fear compared with regular patients, where special instruments have been used [25]. The relationship between caries activity and dental fear is unclear. Further studies are therefore needed examining whether continual caries activity through repeated negative emotions correlates to dental fear.

One of the main findings in our study was the experience expressed in the CA group that they had been treated in a condescending manner and felt disappointed or powerless. This serious expression of negative reception related to caries and dental treatment is an important finding and needs more investigation. Among patients with dental fear, questionnaires with similar questions have been asked such as, “Dentists don't have enough time”, “don't really listen”, “Make me feel guilty” and “Say things to try and fool me” etc. [25].

To the best of our knowledge, there are limited data on experiences of caries active patients. These may be similar to the expressed frustration and a tendency to give up that have been found among adolescents with high caries risk [9]. Similarly, toothache and decayed and filled teeth have been found to have negative effects on satisfaction with dental care [10]. The disappointment and powerlessness among the CA group probably relates to the previously described results that six out of ten of the individuals in this group reported that they did not become free from caries despite extra prophylaxis efforts, and that they have been caries active for a very long period of time [13]. To some extent this could correspond to the challenge and difficulties to promote oral health in high caries risk children [26].

The strengths of this study are primarily that several of the questions have not been addressed before and relate to areas presumably important to individuals that are caries active. Consequently there are very few studies to which to compare results. This study investigated individuals that were grouped according to caries activity, probably one main reason for the very clear difference between the groups. While there is variability across clinical practices, the partial public coverage of dental care for the adult population in Sweden might narrow the variability and enhance patient’s ability to evaluate dental care. This is just one small sample and thus the results must be interpreted with caution, as further studies in larger samples are needed. Nevertheless, the many years of caries activity in the majority of the caries active individuals are similar to findings from other studies [11, 12].

During the follow-up period of this study, there were no national guidelines for caries prophylaxis in Sweden, but this was accomplished in 2011 [27]. In brief, these recommendations state that when a person has an increased risk of developing caries or shows signs of an active caries disease, the dental care givers should suggest that the patient rinse with a 0.2% NaF solution daily. Another alternative is to offer F varnish application in the clinic. The dental personnel should also advise the patients who have a high and frequent sugar intake, to change their dietary habits.

Basic caries prophylaxis, such as information and recommendations of homecare prophylaxis treatments were significantly higher for the CA compared with the CI group. The alternative to offer F varnish application in the clinic was frequently used during the whole follow-up period, and consisted of about half of all basic caries prophylaxis activities in the dental office. However, it was offered equally to both groups, not just to the CA group. If the proposed high risk recommendations in the national guidelines were followed, there would be an annual increase of prophylaxis visits from 0.6 to 4 for each high risk patient.

To decrease problems and negative experiences related to caries and dental treatment among caries active individuals, improvement of caries prophylaxis methods are needed as a prime target. Among dentists there are different preferences for caries prevention; dentists who most frequently use caries prevention seems to be those who also perform caries risk assessment [28]. When caries risk assessment is used, high-risk patients receive more caries preventive recommendations but not to the extent of national guidelines. Improvement of guidelines adherence is needed to stop caries progression [12, 15]. Caries risk assessment using other predictive factors than previous caries experience are therefore needed but yet not available [12, 15, 29]. Patients seem to value dentists who make them aware of existing preventi**v**e options about how to maintain a healthy mouth and teeth [30]; this seems independent from individual caries activity [15].

To improve caries prophylaxis methods international collaborations are needed [31], as well as national attempts to follow-up and develop best practices [32]. In order to help dentists change their practices towards preventive care, it is important to intervene in local networks and to find committed local dental opinion leaders [33]. It will require considerable effort, for dental practices to implement prevention as their clinical norm [34].

## Conclusions

In the present study, caries active individuals reported significantly more problems and negative experiences related to caries and dental treatment. In order to decrease problems and negative experiences, improvement of caries prophylaxis methods are needed.

## Additional file

Additional file 1: Questionnaire used is uploaded as supplementary files: Oral health and caries.pdf. (PDF 173 kb)

## Abbreviations

CA: Caries active
CI: Caries inactive
DMFS: Decayed, missing and filled tooth surfaces
DT: Decayed teeth
KMO: Kaiser-Mayer-Olkin
VAS: Visual analog scale
