# Supplementary material for: Patient-reported negative experiences related to caries and its treatment among Swedish adult patients
Source: BMC Oral Health. 2017 Jun 5;17:95. doi: 10.1186/s12903-017-0384-3 (PMC5460446; doi:10.1186/s12903-017-0384-3)
Supplement: Additional file 1: — Questionnaire used is uploaded as supplementary files: Oral health and caries.pdf. (PDF 173 kb) [file 12903_2017_384_MOESM1_ESM.pdf]

Code number:

Oral health and caries

An investigation about health and dental care

LANDSTINGET VÄSTMANLAND

Karolinska Institutet

Centrum för klinisk forskning
Västerås

Odontologiska Institutionen
Karolinska Institutet, Huddinge

SOME FACTS ABOUT THE STUDY

How is the study conducted?

- The questionanrie have been sent out to a total of 200 patients who have had their salivary flow measured at Folktandvården.
- The results from the questionnaires will be analyzed.
- Results of the investigation will be published.

Why should you fill out this questionnaire?

The reason for this investigation is to find out how: You and others experience your oral health and the caries prophylaxis advice and treatments you may have received.
Your information is important for developing treatments and better care for patients with caries.
In order to achieve valid and generalizable results, we are eager to receive as many responses as possible.
For that reason, your participation is of great value.

Can anyone see your answers?

No. Your answers are not traceable to you.
During the collection of questionnaires a special number code will be used for each questionnaire. This is to check that we have received all answers and sent a reminder if you have forgotten us. The code will be removed as soon as data collection is completed and before the analysis begins.

Do you have any questions?

If you want to know more about the investigation or the questionnaire, you are welcome to contact:

How to fill out the questionnaire

It will take approximately 15 minutes to answer the questions.
It is important that the answers are filled in the right way.

- Use a good pen, black or dark blue. Avoid pencils.
- Put a distinct cross in the squares that you think correspond best for you.
- If you accidentally put a cross in the wrong square, you need to fill the complete square with color. Thereafter put a new cross in the right square.

Your answer

We request that you answer as soon as possible, preferably within a couple of days.

Addressed envelope free of postage

When you have completed the questionnaire, please place it in the enclosed, postage-paid envelope and drop it in any mailbox.

How do you learn about the results?

The results from the study will be published in scientific journals.
A summary will be sent out to all participants.

THANK YOU FOR PARTICIPATION

Håkan Flink, Tandläkare, Folktandvården, Sala
Tel: 0224-580 60 eller 070-68 58 176, e-post: hakan.flink@ltv.se

Åke Tegelberg, Professor, Centrum för klinisk forskning, Västerås
Tel: 021-17 51 30, e-post: ake.tegelberg@ltv.se

Övriga medverkande:

Folke Lagerlöf, Professor, Odontologiska institutionen, Karolinska Institutet, Huddinge

Judy Arnetz, Docent, Institutionen för folkhälso- och vårdvetenskap, Uppsala Universitet

Code number:

ABOUT YOUR GENERALL HEALTH

1 How do you rate your general health, right now?

☐ Very good
☐ Good
☐ Neither good nor poor
☐ Poor
☐ Very poor

2 If poor or very poor, what do you suffer from?

3 Do you use any medication regularly?

☐ Yes
☐ No

If yes, what medication/s?

Medication that you use regularly

Medication that you use periodically

4 Do you have any chronic illness (more than 6 months), permanent problems after an accident, any reduced ability, function or other chronic health problem?

☐ Yes
☐ No

If yes, what kind of illness or type of problem?

ABOUT YOUR ORAL HEALTH

5 How often <u>during the last year</u> have you experienced the following because of <u>trouble with your teeth, mouth, jaw or dentures:</u>? *(Mark <u>one</u> cross on each line)*

	Very often	Often	Occasion ally	Hardly ever	Never
Had trouble pronouncing any words	☐	☐	☐	☐	☐
Felt that your sense of taste has worsened	☐	☐	☐	☐	☐
Had painful aching in your mouth	☐	☐	☐	☐	☐
Found it uncomfortable to eat any foods	☐	☐	☐	☐	☐
Felt self-conscious	☐	☐	☐	☐	☐
Felt tense	☐	☐	☐	☐	☐
Had a diet that was unsatisfactory	☐	☐	☐	☐	☐
Had to interrupt meals	☐	☐	☐	☐	☐
Found it difficult to relax	☐	☐	☐	☐	☐
Been embarrassed	☐	☐	☐	☐	☐
Been a bit irritable with other people	☐	☐	☐	☐	☐
Difficulty doing your usual daily tasks	☐	☐	☐	☐	☐
Felt that life in general was less satisfying	☐	☐	☐	☐	☐
Been totally unable to function	☐	☐	☐	☐	☐

6 How often <u>during the last year</u> has

	Very often	Often	Occasion ally	Hardly ever	Never
Your mouth felt dry	☐	☐	☐	☐	☐

7 How do you rate your <u>oral health</u>, right now?

☐ Very good
☐ Good
☐ Neither good nor poor
☐ Poor
☐ Very poor

EATING HABITS

8 How often do you usually eat breakfast, lunch, dinner, evening snack or other snacks between meals? *(Mark <u>one</u> cross on eatch line)*

	Every day	4-6 days/week	1-3 days/ week	Seldom or never
Breakfast	☐	☐	☐	☐
Lunch	☐	☐	☐	☐
Dinner	☐	☐	☐	☐
Supper	☐	☐	☐	☐
Other between meals	☐	☐	☐	☐

9 When do you eat?
Mark on the "timeline" when you usually put anything in your mouth.
Mark breakfast (B), lunch (L) dinner (D), evening snack (S) and other meals/snacks(X)
Do not include water and sugar-free products

06.00 12.00 18.00 24.00 06.00

10 How often do you eat vegetables and fruits?
- ☐ More than twice a day
- ☐ Two times per day
- ☐ Once a day
- ☐ Sometimes/seldom
- ☐ Never

11 How often do you eat candy/sweets/pastries?
- ☐ More than twice a day
- ☐ Two times per day
- ☐ Once a day
- ☐ Sometimes/seldom
- ☐ Never

12 How often do you drink soft drinks?
- ☐ More than twice a day
- ☐ Two times per day
- ☐ Once a day
- ☐ Sometimes/seldom
- ☐ Never

ORAL HYGIENE

13 How often do you brush your teeth?
- ☐ More than twice a day
- ☐ Two times per day
- ☐ Once a day
- ☐ Sometimes/seldom
- ☐ Never

14 How often do you use dental floss?
- ☐ More than twice a day
- ☐ Two times per day
- ☐ Once a day
- ☐ Sometimes/seldom
- ☐ Never

15 How often do you use toothpicks or proximal brushes?
- ☐ More than twice a day
- ☐ Two times per day
- ☐ Once a day
- ☐ Sometimes/seldom
- ☐ Never

FLUORIDE

16 How often do you use fluoride toothpaste?
- ☐ More than twice a day
- ☐ Two times per day
- ☐ Once a day
- ☐ Sometimes/seldom
- ☐ Never

17 How often do you use any kind of extra fluoride, tablets, chewing gum or rinses?
- ☐ More than twice a day
- ☐ Two times per day
- ☐ Once a day
- ☐ Sometimes/seldom
- ☐ Never

YOUR DENTAL CARE EXPERIENCE

18 How often have you, as an adult, experienced the following during visits for dental treatment?
(Mark one cross on each line)

	Very often	Often	Occasionally	Hardly ever	Never
Told you had caries	☐	☐	☐	☐	☐
Your dentist told you needed extra caries-preventive treatment	☐	☐	☐	☐	☐
Informed about causes of caries	☐	☐	☐	☐	☐
Recommended to reduce the number of snacks between meals	☐	☐	☐	☐	☐
Recommended limiting your intake of sugar	☐	☐	☐	☐	☐
Recommended using some form of extra fluoride, for example tablets, chewing gum or rinses	☐	☐	☐	☐	☐
Had your teeth polished	☐	☐	☐	☐	☐
Had fluoride varnish applied to your teeth	☐	☐	☐	☐	☐
Instructed how to brush your teeth	☐	☐	☐	☐	☐
Instructed how to use dental floss or other devices to clean between your teeth	☐	☐	☐	☐	☐
Told your immunity to caries was impaired	☐	☐	☐	☐	☐
Treatment was painful	☐	☐	☐	☐	☐
You felt calm	☐	☐	☐	☐	☐
You felt frightened	☐	☐	☐	☐	☐
You felt stressed	☐	☐	☐	☐	☐
You felt anxious	☐	☐	☐	☐	☐
You felt you were in control	☐	☐	☐	☐	☐
You were treated in a condescending manner	☐	☐	☐	☐	☐
You were disappointed	☐	☐	☐	☐	☐
You felt powerless	☐	☐	☐	☐	☐

19 What is your opinion about the following statements?

(Mark one cross on each line)	Agree completely	Agree fairly well	Do not agree completely	Do not agree at all
You have suspected your defense for caries has been reduced	☐	☐	☐	☐
Dental staff have listened to your problems/needs	☐	☐	☐	☐
Dental staff have not believed that you made enough prophylaxis efforts	☐	☐	☐	☐
Dental staff have believed that you were not working hard to avoid caries	☐	☐	☐	☐

PROFYLACTIC TREATMENT AGAINST CARIES

20 How often as an adult have you made extra efforts to avoid caries, by ……….

(Mark one cross on each line)

	Very often	Often	Occasion ally	Hardly ever	Never
… *Changing to better eating habits*, not eat frequently, less sugar, more fruits and vegetables	☐	☐	☐	☐	☐
… *Performing better oral cleaning*, tooth brushing, dental floss and other aids for cleaning in between teeth	☐	☐	☐	☐	☐
… *Use of extra fluoride*, for example tablets, chewing gum or rinses	☐	☐	☐	☐	☐

RESULT OF PROFYLACTIC TREATMENT AGAINST CARIES

21 What is your opinion about the extra prophylaxis measures mentioned in question 20?

(Mark one cross on each line)

	Agree completely	Agree fairly well	Do not agree completely	Do not agree at all	Not applicable
Extra prophylaxis efforts have been easy to perform	☐	☐	☐	☐	☐
Extra prophylaxis efforts have made me free from caries (not needing fillings)	☐	☐	☐	☐	☐
I am pleased with the results of the extra prophylaxis efforts	☐	☐	☐	☐	☐
I have not been in need of extra prophylaxis efforts, I seldom or never get caries	☐	☐	☐	☐	☐
I have had difficulty remembering to perform the extra prophylaxis efforts	☐	☐	☐	☐	☐

VALUES AND EXPECTATIONS

22 What is our opinion about the following statements?

(Mark one cross on eatch line)

	Agree completely	Agree fairly well	Do not agree completely	Do not agree at all
With prophylaxis efforts it is possible for me to become free from caries	☐	☐	☐	☐
I place great value on efforts that reduce caries cavities in my teeth	☐	☐	☐	☐
I would spend more time with home care if I knew it would reduce caries cavities in my teeth	☐	☐	☐	☐

23 What was your perception about caries before the saliva test was taken

(Put a vertical line on the horizontal line below, that corresponds to the degree of your problem)

	Very much a problem	No problem
Caries was problem for me	●—————————————	●

Continue to next page ➢➢

23 What was your perception about caries before the saliva test was taken

(Mark one cross on each line)	Agree completely	Agree fairly well	Do not agree completely	Do not agree at all
Caries was a problem for my *economic* situation	☐	☐	☐	☐
The *time* I spent at the dentist for caries treatment has been a problem for me	☐	☐	☐	☐
The *discomfort during treatment* at the dentist was a problem for me	☐	☐	☐	☐
Caries causing *trouble/pain* from my teeth was a problem for me	☐	☐	☐	☐

24 What is your perception about caries today
(Put a vertical line on the horizontal line below, that corresponds to the degree of your problem)

	Very much a problem		No problem
Caries is a problem for me	●——————————————————————●		

(Mark one cross on each line)	Agree completely	Agree fairly well	Do not agree completely	Do not agree at all
Caries is a problem for my *economic* situation	☐	☐	☐	☐
The *time* I spend at the dentist for caries treatment is a problem for me	☐	☐	☐	☐
The *discomfort during treatment* at the dentist is a problem for me	☐	☐	☐	☐
Caries causing *trouble/pain* from my teeth is a problem for me	☐	☐	☐	☐

25 What is most important for you if you want to avoid caries?
Rank the alternatives in order of preference from 1-5, where 1 is most important, using each number once.

That my cost for dental treatment will be reduced	
That I save time by fewer dental appointments	
That unpleasant dental treatments will be reduced	
That trouble/pain from my teeth will be reduced	
That I avoid fillings in my teeth	

26 How much would you be willing to pay per month for a prophylaxis method that makes you free from caries? (one filling today costs approximately 800 skr=swedish crowns)

☐ 0 skr/month
☐ 50 skr/month
☐ 100 skr/month
☐ 150 skr/month
☐ More than 200 skr/month

EDUCATION / WORK / ECONOMY

27 What is your highest level of education?

☐ Elementary school
☐ High school
☐ College or University

28 How high is your yearly income, right now?

☐ Less than 100 000 skr
☐ 100 000 – 150 000 skr
☐ 151 000 – 200 000 skr
☐ 201 000 – 250 000 skr
☐ 251 000 – 300 000 skr
☐ 301 000 – 400 000 skr
☐ More than 400 000 skr

29 How high is your family yearly income, right now?

☐ Less than 100 000 skr
☐ 100 000 – 200 000 skr
☐ 201 000 – 300 000 skr
☐ 301 000 – 400 000 skr
☐ 401 000 – 500 000 skr
☐ 501 000 – 600 000 skr
☐ More than 600 000 skr

30 Do you work

☐ Daytime
☐ Night
☐ Mixed day and night
☐ Do not work – sick leave/retired
☐ Do not work – job seeker/student

31 During the last 12 months have you had difficulties to pay rent or other bills?

☐ No
☐ Yes, 1 month
☐ Yes, 2 months
☐ Yes, 3-5 months
☐ Yes, 6-12 months

32 How has your financial situation been as an adult?

☐ Very good
☐ Good
☐ Neither good nor bad
☐ Bad
☐ Very bad

OTHER

33 Do you smoke?

☐ No, I have never smoked
☐ No, I have stopped
☐ Yes, I smoke occasionally
☐ Yes, I smoke daily

34 How do you sleep?
(more than one alternative can be marked)

☐ Normal sleep - thoroughly rested
☐ Disturbed sleep – not thoroughly rested
☐ Disturbed sleep – problem falling asleep at night
☐ Disturbed sleep – waking during the night

If disturbed sleep, how many months?

☐ Month(s)

35 Are you?

☐ Female
☐ Male

36 What year were you born?

19 ☐

37 How much do you weigh? *(whole kilogram)*

I weight ☐ kilograms

38 How tall are you? *(Whole cm)*

I am ☐ cm tall

Thank you very much for your participation!

Mail the filled questionnaire by the enclosed envelope free of postage. No stamps needed.

YOUR OWN ADDITIONAL COMMENTS

....

....

....

....

....

....

....

....

....

....

....

....

....

....

....
